# Antibody Responses to SARS-CoV-2 Antigens in Humans and Animals

**DOI:** 10.3390/vaccines8040684

**Published:** 2020-11-16

**Authors:** Hyunsuh Kim, Patrick Seiler, Jeremy C. Jones, Granger Ridout, Kristi P. Camp, Thomas P. Fabrizio, Trushar Jeevan, Lance A. Miller, Robert E. Throm, Francesca Ferrara, Richard L. Fredrickson, James F. Lowe, Leyi Wang, Solomon O. Odemuyiwa, Xiu-Feng Wan, Richard J. Webby

**Affiliations:** 1 Department of Infectious Diseases, St. Jude Children’s Research Hospital, Memphis, TN 38105, USA; Hyunsuh.Kim@stjude.org (H.K.); Jon.Seiler@stjude.org (P.S.); Jeremy.Jones@stjude.org (J.C.J.); Thomas.Fabrizio@stjude.org (T.P.F.); Trushar.Jeevan@stjude.org (T.J.); Lance.Miller@stjude.org (L.A.M.); 2 Hartwell Center for Bioinformatics & Biotechnology, St. Jude Children’s Research Hospital, Memphis, TN 38105, USA; Granger.Ridout@stjude.org; 3 Eastgate Animal Clinic, Memphis, TN 38117, USA; kcamp@vetmail.lsu.edu; 4 Vector Development & Production, St. Jude Children’s Research Hospital, Memphis, TN 38105, USA; Robert.Throm@stjude.org (R.E.T.); Francesca.Ferrara@stjude.org (F.F.); 5 Veterinary Diagnostic Laboratory, College of Veterinary Medicine, University of Illinois at Urbana-Champaign, Urbana, IL 61820, USA; frdrcksn@illinois.edu; 6 Integrated Food Animal Management Systems, Department of Veterinary Clinical Medicine, College of Veterinary Medicine, University of Illinois at Urbana-Champaign, Urbana, IL 61820, USA; jlowe@illinois.edu; 7 Department of Veterinary Clinical Medicine and the Veterinary Diagnostic Laboratory, College of Veterinary Medicine, University of Illinois at Urbana-Champaign, Urbana, IL 61820, USA; leyiwang@illinois.edu; 8 Department of Veterinary Pathobiology, College of Veterinary Medicine, University of Missouri, Columbia, MO 65211, USA; odemuyiwas@missouri.edu (S.O.O.); wanx@missouri.edu (X.-F.W.); 9 Department of Microbiology, Immunology & Biochemistry, College of Medicine, The University of Tennessee Health Science Center, Memphis, TN 38163, USA

**Keywords:** SARS-CoV-2, COVID-19, respiratory viruses, antibody, vaccine

## Abstract

To optimize the public health response to coronavirus disease 2019 (COVID-19), we must first understand the antibody response to individual proteins on the severe acute respiratory syndrome-related coronavirus 2 (SARS-CoV-2) and the antibody’s cross reactivity to other coronaviruses. Using a panel of 37 convalescent COVID-19 human serum samples, we showed that the magnitude and specificity of responses varied across individuals, independent of their reactivity to seasonal human coronaviruses (HCoVs). These data suggest that COVID-19 vaccines will elicit primary humoral immune responses in naïve individuals and variable responses in those previously exposed to SARS-CoV-2. Unlike the limited cross-coronavirus reactivities in humans, serum samples from 96 dogs and 10 cats showed SARS-CoV-2 protein-specific responses focused on non–S1 proteins. The correlation of this response with those to other coronaviruses suggests that the antibodies are cross-reactive and generated to endemic viruses within these hosts, which must be considered in seroepidemiologic studies. We conclude that substantial variation in antibody generation against coronavirus proteins will influence interpretations of serologic data in the clinical and veterinary settings.

## 1. Introduction

The emergence of the COVID-19 pandemic motivated a remarkable global effort to understand the virus that caused it, SARS-CoV-2, to elucidate the responses of humans to infection, and to develop potential vaccination strategies. The latter 2 elements are very much intertwined because understanding the human antibody response to SARS-CoV-2 infection is important for optimal vaccine design. Although this area has been a research priority and the focus of numerous studies, much uncertainty remains.

Despite the presence of multiple endemic coronaviruses in humans (i.e., 229E, NL63, OC43, and HKU1), unexposed individuals have very low-to-no preexisting antibody responses to SARS-CoV-2 [1,2], suggesting that the functional cross reactivity between the endemic viruses and the pandemic virus is limited. Upon SARS-CoV-2 infection, however, robust and rapid antibody responses are generated in most symptomatic individuals [3,4,5,6]. Immune responses to the viral proteins, spike (S) (including its subdomains S1, S2, and the receptor-binding domain [RBD]) and nucleoprotein (N), as determined by ELISA, have been the most extensively studied [3,4,5], with titers to RBD showing the best correlation with neutralizing responses [5]. Although the duration of these responses has yet to be determined, a report has suggested that the antibody levels drop soon after peak titers have been reached [7]. If so, this brief response raises concerns for successful vaccine development.

Vaccine development against SARS-CoV-2 has proceeded at an unprecedented rate since the virus emerged, with multiple platforms under consideration and evaluation reviewed in [8]. Prior studies with SARS-CoV showed that antibodies targeting S are extremely effective at neutralizing the virus and provide protection in animal models [9,10]. Based on that work and results from other coronavirus studies, most early SARS-CoV-2 vaccine platforms under development target S as their major mechanism of action. Although such approaches are appropriate, new strategies also must be explored if S-based platforms underperform or enhance disease, as has been observed for other coronaviruses including SARS-CoV [11,12]. These next-generation vaccine strategies are best informed by a better understanding of post-infection antibody response to different SARS-CoV-2 proteins developed under natural conditions, including evaluation of interpersonal variability.

Evaluating preexisting and convalescent antibody responses is also important for correctly interpreting seroepidemiologic studies of SARS-CoV-2 and its related viruses. These studies are crucial for global public health responses, and they aid in generating estimates of true infection rates in humans. Furthermore, these studies may provide evidence for the potential role of animal hosts, particularly companion animals such as dogs and cats [12,13,14], in the future ecology of the disease. Such studies are complicated by the presence of endemic coronaviruses and would benefit from inclusion of non-SARS-CoV-2 antibody analyses in each host.

In this report, we examine the antibody responses to SARS-CoV-2 proteins after natural infection and immunization, and we further explore the antigenic cross reactivity of the virus with endemic coronaviruses of humans, dogs, and cats.

## 2. Materials and Methods

### 2.1. Human Serum Samples

All human sera samples were obtained from commercial sources BioIVT (Westbury, NY, USA) or BioChemed Services (Winchester, VA, USA). The samples were collected 17–30 days after symptom onset from 35 patients who were SARS-CoV-2-positive by real-time polymerase chain reaction (RT-PCR) analysis and had mild-to-moderate COVID-19 disease. Sera were collected at 18– and 19–days post-test in 2 asymptomatic carriers who had positive RT-PCR test results. Pulmonary involvement was demonstrated in 1 patient, and 1 hospitalized patient with cancer was included. The cohort included 20 female and 17 male patients; their mean age was 46 years (range, 24–77 years). Serum samples from 9 healthy, COVID-19-negative individuals that were obtained before December 31, 2015 were used as negative controls. All procedures performed in studies involving human participants were in accordance with the ethical standards of the institutional and/or national research committee and with the 1964 Helsinki Declaration and its later amendments or comparable ethical standards.

### 2.2. Dog and Cat Serum Samples

Serum samples were collected from 96 dogs and 10 cats at a veterinary clinic in Memphis, TN, and represented a convenience sample set. None of the animals were vaccinated for canine coronavirus (CCoV) or feline coronavirus (FCoV), and all were SARS-CoV-2-negative at time of blood collection. Convalescent sera from 2 cats infected with feline infectious peritonitis (FIP) were available. Sera samples from specific pathogen-free (SPF) dogs (*n* = 5) and cats (*n* = 3) were obtained from Marshall BioResources (North Rose, NY, USA) and used as negative controls.

### 2.3. Antigen Sources

Antigens from several sources were used for immunization and ELISA experiments. SARS-CoV-2 envelope (E), RBD, and S were sourced from ABclonal Inc. (Woburn, MA, USA); N, S1, and S2, from Sino Biological Inc. (Beijing, China); and nonstructural protein 3 (NS3), from antibodies-online Inc. (Dunwoody, GA, USA). SARS-CoV S was sourced from eENZYME LLC (Gaithersburg, MD, USA); SARS-CoV E, matrix (M), N, and 3C-like protease (3CLpro) were obtained from BEI Resources (NIAID, NIH, Bethesda, MD, USA). SARS-related coronavirus 2, isolate USA-WA1/2020, and γ-irradiated SARS-CoV, deposited by the Centers for Disease Control and Prevention, were also sourced from BEI Resources.

### 2.4. Immunization of Mice

Seven-week-old BALB/c mice (Jackson Laboratory, Bar Harbor, ME, USA) were immunized via the intramuscular (i.m.) route with 2 doses (5 µg/dose) of recombinant protein (i.e., S, S1, RBD, S2, N, E, or NS3) of SARS-CoV-2, inactivated whole virus of SARS-CoV-2, or recombinant protein of SARS-CoV (i.e., S, N, E, M, or 3CLpro). At day 24 after the second immunization, blood, spinal bone marrow (BM), and cervical lymph nodes (LNs) were collected from the mice. The experiments were performed in accordance with the guidelines set by the Animal Care and Use Committee of St. Jude Children’s Research Hospital in an enhanced Animal Biosafety Level-2 containment facility. The activities were registered under protocol 428 approved on August 12 2020. 

### 2.5. ELISA

Levels of IgG, IgA, and IgM antibodies in human sera and animal sera were measured by an indirect ELISA with recombinant proteins (S, S1, RBD, S2, N, E, and NS3 of SARS-CoV-2 and S, N, E, M, and 3CLpro of SARS-CoV), and whole inactivated virus antigens (SARS-CoV-2, HCoV NL63, CCoV 1-71, and H1N1 influenza virus [A/California/2009/07]), which were prepared by density-gradient purification, followed by γ-irradiation or β-propiolactone inactivation. Heat-inactivated sera (56 °C for 30 min) were added to the wells of antigen-coated plates and then detected by the respective horseradish peroxidase (HRP)-conjugated secondary antibodies (SouthernBiotech, Birmingham, AL, USA). TMB substrate (MilliporeSigma, Burlington, MA, USA) was added to produce a color change. The reaction was stopped with sulfuric acid, and optical density (OD) at 450 nm was read using the Synergy plate reader (Biotek, Broadview, IL, USA). The cutoff values of tests were defined as 3 times the average of the negative control samples for human sera, 3 times the average of the SPF samples for canine and feline sera, and 3 times the average of the preimmune murine sera.

### 2.6. Protein Microarray Assays

Antigens printed onto microarrays were glycoproteins or nucleoproteins of HCoVs, Middle East respiratory syndrome coronavirus (MERS-CoV), SARS-CoV, SARS-CoV-2, respiratory syncytial viruses (RSVs), metapneumoviruses (MPVs), parainfluenza viruses (PIVs), adenoviruses (AdVs), and influenza viruses. Protein microarray assays were performed according to the manufacturer’s instructions (Sino Biological, Beijing, China). Briefly, slides were probed with human sera in protein-blocking buffer overnight at 4 °C and labeled with Alexa Fluor^®^ 647 anti-human IgG (Jackson ImmunoResearch, West Grove, PA, USA) to quantum dot fluorophore for 1 h at room temperature. Dried slides were imaged using the GenePix 4000B microarray scanner (Molecular Devices, San Jose, CA, USA). Mean fluorescence intensity (MFI) of the 4 replicate spots for each antigen was used for the analysis.

### 2.7. Microneutralization Assays

Equal volumes of SARS-CoV-2 S-pseudotyped lentivirus and serial dilutions of sera were incubated for 1 h at room temperature, and then virus–serum mixtures were added to VeroE6/TMPSS2 cell plates (Sekisui XenoTech, Kansas City, KS, USA). After 48 h, 100 µL Bright-Glo luciferase assay reagent (Promega, Madison, WI, USA) was added to each well. The entire 200-µL volume was then transferred to Corning costar white opaque 96-well assay plates (Corning, NY, USA), and the luminescent output was read in a Synergy plate reader (Biotek, Broadview, IL, USA). Inhibition curves were prepared and IC_50_ values were determined by nonlinear regression analyses in Graph Pad Prism 8 (GraphPad Software, San Diego, CA, USA) using the [log_2_] vs inhibitor, 4-point sigmoidal equation.

### 2.8. ELISpot

The spinal BM cells from the immunized mice were isolated after red blood cells were lysed via incubation with ammonium-chloride-potassium-lysing buffer (Lonza, Basel, Switzerland). BM plasma cells (PCs) were isolated by magnetic labeling using the Plasma Cell Isolation Kit (Miltenyi Biotec, Auburn, CA, USA). Cervical LN B cells of mice were prepared by depletion of non-B cells using the B Cell Isolation Kit (Miltenyi Biotec, Auburn, CA, USA) followed by homogenization using gentleMACS C tubes (Miltenyi Biotec, Auburn, CA, USA). Of the remaining population, 10^5^ live BM and LN B cells were apportioned to antigen-coated 96-well plates (Millipore, Darmstadt, Germany), and then the plates were incubated at 37 °C for 4 h. The plates were then washed with PBS and 0.5% Tween in PBS, and PCs and antibody-secreting cells (ASCs) were detected by anti–mouse IgG HRP secondary antibody (SouthernBiotech, Birmingham, AL, USA). Spots were developed by the chromogen substrate AEC (3-amino-9-ethylcarbazole; Sigma-Aldrich, St. Louis, MO, USA) and water was added to stop the reaction. Spots were analyzed by an Immunospot analyzer (Cellular Technology Limited, Shaker Heights, OH, USA).

## 3. Results

### 3.1. Human Peripheral Antibody Responses to SARS-CoV-2 Proteins

Of the 37 serum samples collected from patients with laboratory-confirmed COVID-19 disease, 2 (5.4%) were from asymptomatic carriers, 34 (91.9%) were from patients who showed mild-to-moderate symptoms, and 1 (2.7%) was from a hospitalized patient. Serum samples were collected 17–30 days after symptom onset or at 18 or 19 days after the COVID-19-positive test in asymptomatic individuals. Serum samples from the control group were also tested. 

**Figure 1:**
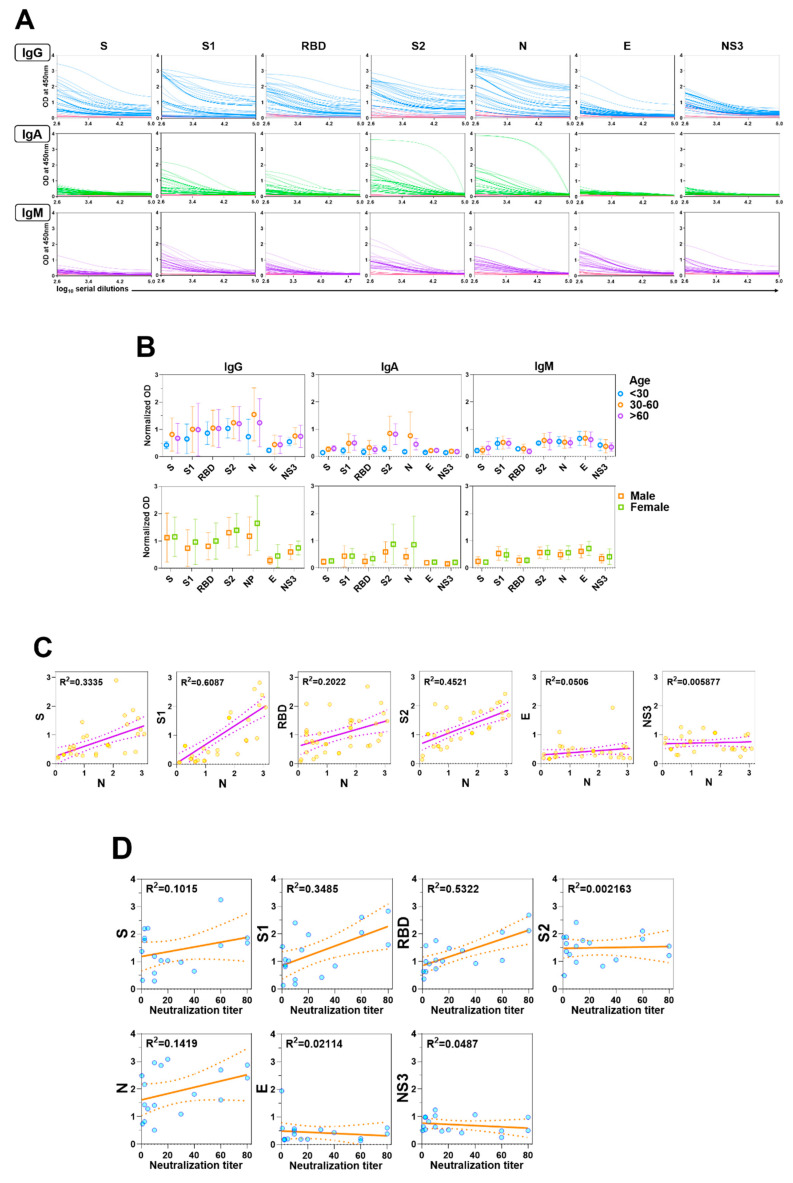
Antibody responses in patients with COVID-19. (**A**) Levels of serum IgG, IgA, and IgM antibodies against S, S1, RBD, S2, N, E, and NS3 of SARS-CoV-2 were measured by ELISA. Blue (IgG), green (IgA), and purple (IgM) lines represent the antibody titers of individual COVID-19 positive sera, pink lines represent negative control sera. (**B**) Each dot represents an individual antibody titer, and groups are divided by age and sex. (**C**) Correlation of anti-N responses with other antiviral protein responses in patients with COVID-19. Correlations of the values were assessed by linear regression. (**D**) Correlations between neutralization titer (x-axis) and each antibody response to the viral protein of SARS-CoV-2 (y-axis) are shown. Each dot indicates the neutralization titer of serum antibodies of patients with COVID-19, as determined by calculating the highest dilution of serum that prevents infection of 50% of inoculations and antiviral protein titers (IgG) of human sera, as determined by ELISA.

Considerable data have been published on human responses to S and N viral proteins, but less is known about responses to other SARS-CoV-2 proteins. We measured serum IgG, IgA, and IgM levels in response to the SARS-CoV-2 proteins, S, S1, RBD, S2, N, E, and NS3 in patients with COVID-19. IgG responses (as determined relative to negative control responses) to S, S1, RBD, S2, and N were detected in all of the patient samples (Figure 1A). Two individuals (both with mild-to-moderate disease) had no responses to E, and low responses to S and/or N. The IgG responses of all responders to E and NS3 were lower than those to the other viral proteins (Figure 1A). The relative responses to each protein varied across patients; for example, some responded more vigorously to S2 than to other proteins, while others responded more vigorously to N. This is highlighted by the irregular correlation of N antibody levels with other SARS-CoV-2 proteins (Figure 1C), including the lack of a consistent pattern between N and S reactivities. Similarly, the response to the S subdomains (S1, S2, and RBD) varied considerably across individuals. We saw no reactivity to the SARS-CoV-2 proteins in the negative controls (Figure 1A). In general, IgA and IgM responses were lower than IgG responses (Figure 1A). We detected no significant differences in antibody responses to the SARS-CoV-2 proteins based on age or sex (Figure 1B).

### 3.2. Human Neutralizing Antibodies Against SARS-CoV-2

Despite the wide variety of COVID-19 infection-induced antibodies, only some serve as barriers to viral infection. To determine which viral protein-specific antibody titers correlated most strongly with neutralization, we evaluated the neutralizing titers of the human sera with a SARS-CoV-2 S-pseudotyped lentivirus on VeroE6/TMPSS2 cells. We then compared these results with the IgG ELISA titers in response to S, S1, RBD, S2, N, E, and NS3 as determined above (Figure 1A).

Overall, 17 of the 37 (45.9%) samples showed some level of neutralizing activity, though the strength of the responses was considerably diverse. The strongest correlation to neutralizing antibody titers among the 17 sera samples was in RBD-specific ELISA antibody titers (95% CI, 0.007782–0.02438; R^2^ = 0.5322) (Figure 1D). We found only a minimal correlation with ELISA antibody titers of the other viral proteins, including S1 and S (both containing RBD; S1, R^2^ = 0.3485; S, R^2^ = 0.1015) (Figure 1D).

### 3.3. Cross reactivity of SARS-CoV-2 antibodies to other human coronaviruses

**Figure 2:**
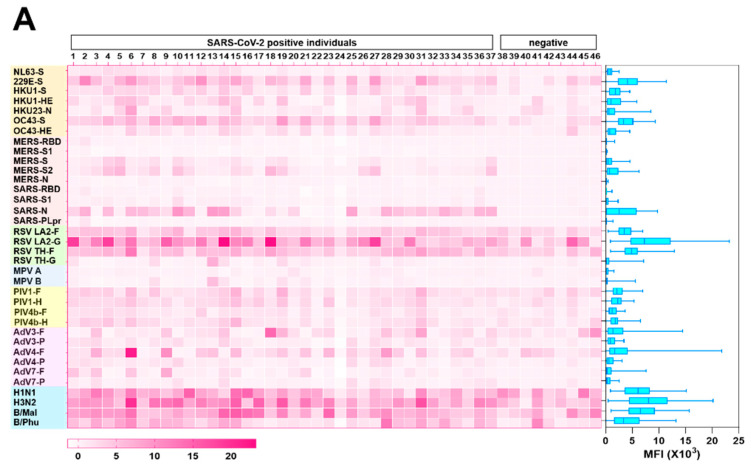

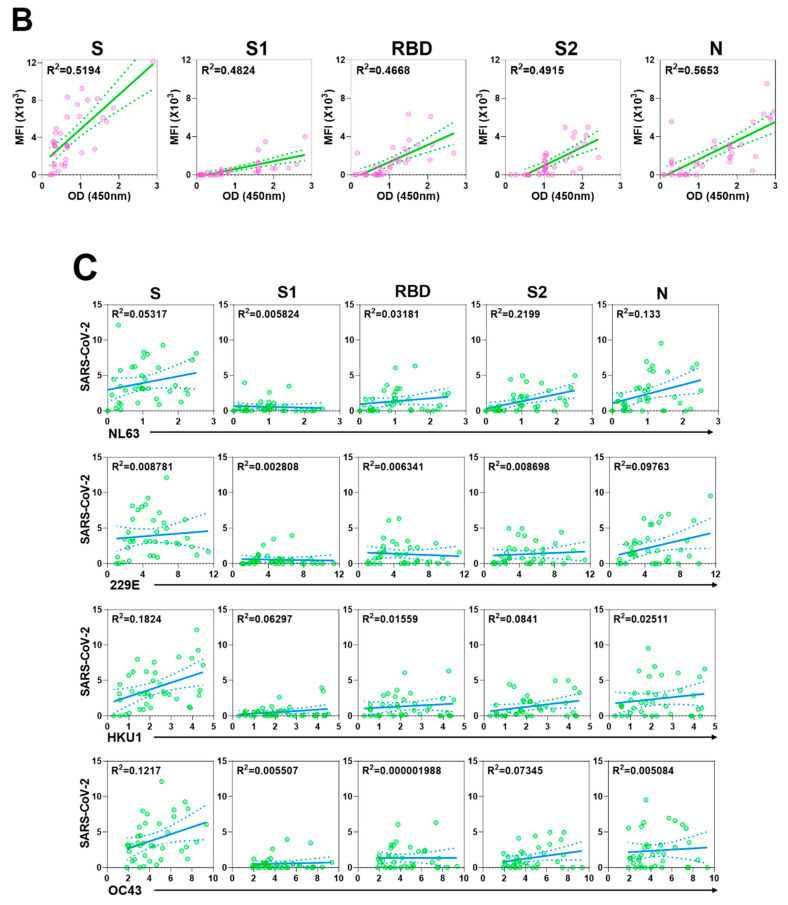
Preexisting and cross-reactive antibodies to human respiratory viruses in sera of patients with COVID-19. (**A**) Respiratory virus-specific antibody profiling. Heatmap indicating IgG antibody responses in 46 sera samples (37 COVID-19 samples and 9 control samples) measured by protein microarray. The microarray slides were probed with human sera and labeled with secondary antibodies to human IgG conjugated to a quantum dot fluorophore. The slides were imaged using the GenePix 4000B Microarray Scanner to measure background-subtracted median spot fluorescence. Mean fluorescence intensity (MFI) of the 4 replicates for each antigen was used for analysis. Each column represents a paired sample, and each row, a respiratory virus protein. Box graph indicates the sum of antibody responses of all sera samples for each respiratory virus protein. (**B**) Correlation between protein microarray and ELISA-based readouts. Each dot indicates the antibody response of serum from a single patient with COVID-19 against S, S1, RBD, S2, and N of SARS-CoV-2, as measured by fluorescence intensity (y-axis) and absorbance at 450 nm, as determined by ELISA (x-axis). (**C**) Relations of antibody response between specific antibody levels against SARS-CoV-2 and those against endemic HCoVs NL63, 229E, HKU1, and OC43 in patients with COVID-19.

Next, we examined the reactivity of the convalescent SARS-CoV-2 sera to S proteins of endemic HCoVs, 229E, NL63, OC43, and HKU1 by using a protein microarray to determine whether the magnitude of antibody responses to SARS-CoV-2 is influenced by prior coronavirus exposure (Figure 2A). First, to ensure the robustness of the protein microarray data, we compared titers to S, S1, RBD, S2, and N proteins of SARS-CoV-2, as measured by ELISA and by protein microarray. In general, there was good agreement between the two measures, thereby demonstrating the accuracy of the microarray platform (Figure 2B).

We detected abundant IgG antibodies to the S proteins of HCoVs in the sera of patients with COVID-19. The correlation between antibody levels to the S proteins of HCoVs and those to viral proteins of SARS-CoV-2 was minimal. Similarly, the magnitude of antibody responses to SARS-CoV-2 S was not related to HCoVs’ S titers, which suggested there is little-to-no functional cross-reactivity between the viruses (Figure 2C). As demonstrated by others [3,15,16,17], we did note reactivity to viral proteins of MERS-CoV (S and S2) and SARS-CoV (N) in the sera from SARS-CoV-2-infected patients, highlighting the similarity between these viruses (Figure 2A).

### 3.4. Inter-Person Variation in Antibody Levels to Other Respiratory Viruses

A major unanswered question in the context of the antibody response to SARS-CoV-2 is the duration of the response. We were intrigued to determine the levels of antibodies to common respiratory viruses in our human serum samples. We examined antibody responses to those viruses associated with human respiratory disease, including RSVs, MPVs, PIVs, AdVs, and influenza viruses, by protein microarray (Figure 2A). In general, responses were strongest to RSVs and influenza viruses and weakest to MPVs. The low titers to MPV, though nearly everyone has been infected with this virus by the age of 5 years [18], suggests that antibody decline over time reflects the responses to typical respiratory viruses.

### 3.5. Murine Response to SARS-CoV-2 Antigen Immunization

**Figure 3:**
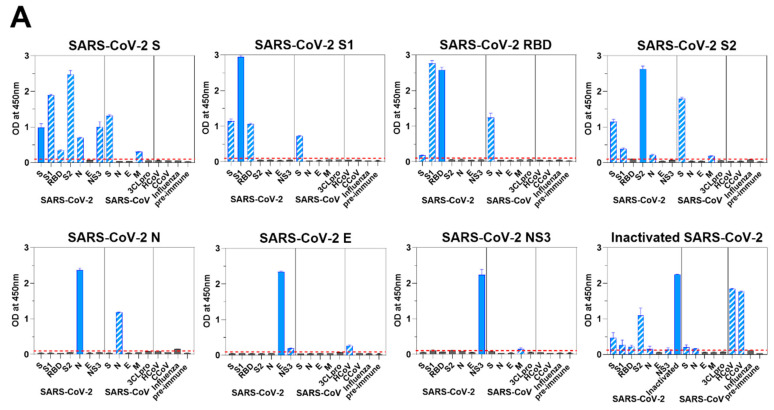

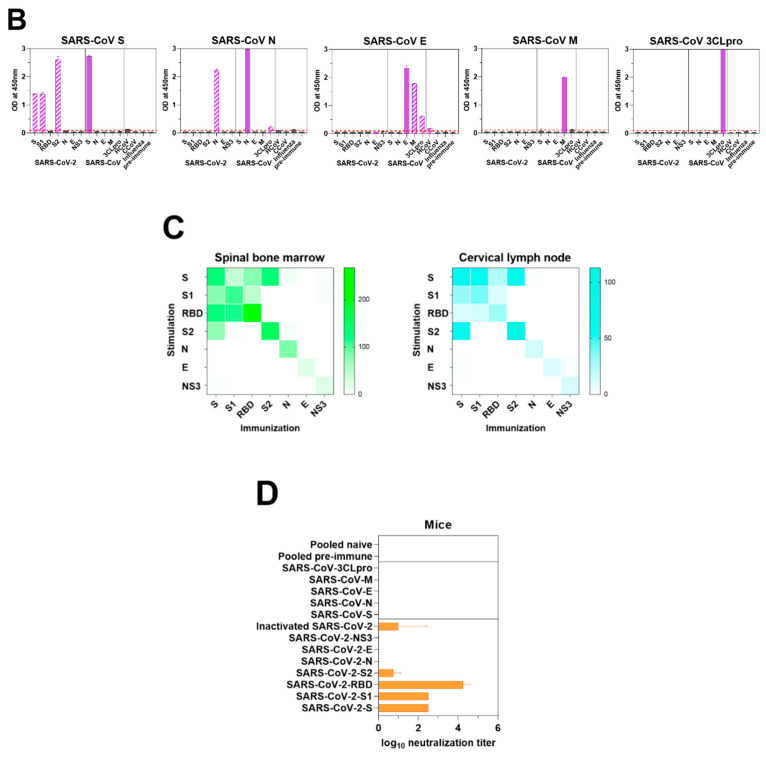
Immune responses of mice to SARS-CoV-2. (**A**) Viral protein-specific IgG antibody responses of mice immunized with S, S1, RBD, S2, N, E, or NS3 of SARS-CoV-2. Viral protein-specific IgG responses of murine sera and cross-reactivities to the viral proteins of SARS-CoV, CCoV 1-71, HCoV NL63, and influenza virus were measured by ELISA. Blue bars represent homologous antibody responses, and bars with slash lines display cross-reactive antibody responses. Red lines are the cut-offs calculated as 3 times the mean absorbance value of the preimmune sera. (**B**) Antibody response was measured in mice immunized with S, N, E, M, or 3CLpro of SARS-CoV. Purple bars show homologous antibody responses, and bars with slash lines represent cross-reactive antibody responses. (**C**) S-, S1-, RBD-, S2-, N-, E-, and NS3-specific IgG PCs in spinal bone marrow (left) and ASCs in cervical lymph nodes (right) were quantified ex vivo in ELISpot assays. Bone marrow and lymph node cells were obtained from the mice immunized with respective viral protein of SARS-CoV-2. (**D**) The presence of neutralizing antibodies in mice immunized with S, S1, RBD, S2, N, E, or NS3 of SARS-CoV-2; inactivated whole virus of SARS-CoV-2; and S, N, E, M, or 3CLpro of SARS-CoV was measured by in vitro neutralization assays using a pseudotyped virus. The transduction efficacy was determined by measuring the luciferase activity. Nonlinear regression analysis was used to obtain the IC_50_ in the neutralization assays.

We hypothesized that the antibody responses provoked by SARS-CoV-2 antigen immunization might have recognizable differences to those made during a natural infection. Therefore, we assessed individual protein-specific responses together with potential coronavirus cross-reactivity in mice that were immunized with SARS-CoV-2 antigens as described above. We used ELISA to assess the murine sera for cross-reactive antibody responses to viral proteins of SARS-CoV-2 (Figure 3A) and SARS-CoV (Figure 3B). In all cases, we saw the highest ELISA titers with the immunizing antigens. As expected, sera from S1-immunized mice reacted with S and RBD; sera from mice immunized with SARS-CoV-2 RBD reacted to SARS-CoV-2 S1, but somewhat unexpectedly, not well to SARS-CoV-2 S; and sera from S2-immunized mice reacted with S (Figure 3A). Immunoreactivities of sera from mice immunized with SARS-CoV-2 S were specifically broad, they reacted to not only S subunits, S1, RBD and S2, but also N and NS3 (Figure 3A).

These trends were mirrored in responding B-cell cross reactivity in both BM and LN cells. We detected high numbers of antigen-specific PCs and ASCs 24 days post–boosting immunization with each protein: spinal BM PCs (N, 94; S, 136; S1, 109; S2, 158; RBD, 267; E, 24; and NS3, 26 per 10^5^ cells) and cervical LN ASCs (N, 16; S, 64; S1, 40; S2, 130; RBD, 29; E, 12; and NS3, 14 per 10^5^ cells) (Figure 3C). 

The correlation between antibodies to the RBD of SARS-CoV-2 and neutralization titers observed in human convalescent sera was also seen in antibody responses elicited by immunization in mice. Neutralization titers of antibodies to the RBD were significantly higher (mean titer=10^4.25^, *p* < 0.0001) than those of antibodies to other viral proteins (Figure 3D). Sera from mice immunized with RBD, S1, and S had neutralizing activity. Antibodies generated from S2 and inactivated whole-virus immunization also neutralized the pseudotyped virus, albeit at low levels (Figure 3D).

Interestingly, although we detected very limited cross reactivity of any of the SARS-CoV-2 protein–specific responses to the endemic HCoV NL63 or CCoV 1-71, immunization of mice with whole inactivated SARS-CoV-2 virions resulted in robust cross-reactive responses to these heterologous coronaviruses (Figure 3A).

### 3.6. Coronavirus Antibodies in Animal Sera

Due to the observed CCoV 1-71 cross-reactivity of murine antisera generated by inactivated SARS-CoV-2 immunization, we next sought to examine the level of coronavirus antibodies in canine serum samples. We screened serum samples collected from 96 dogs with no history of coronavirus vaccination at a veterinary clinic and 5 serum samples collected from SPF dogs (*n* = 101 dogs). These sera were screened for reactivity to CCoV 1-71, HCoV NL63, and influenza A virus (H1N1) by whole-virus antigen ELISA and against the recombinant proteins of SARS-CoV-2.

**Figure 4:**
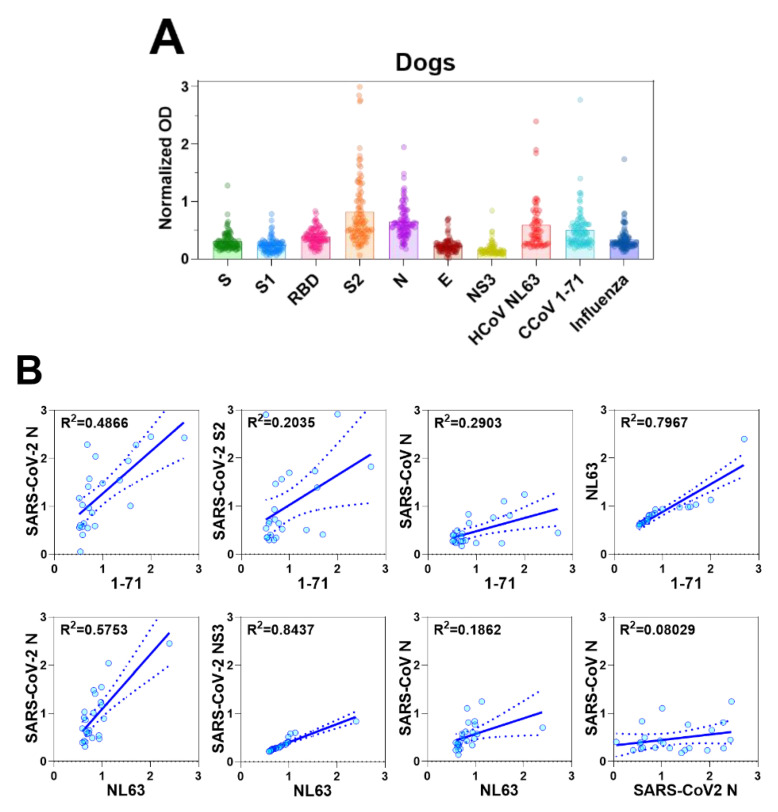

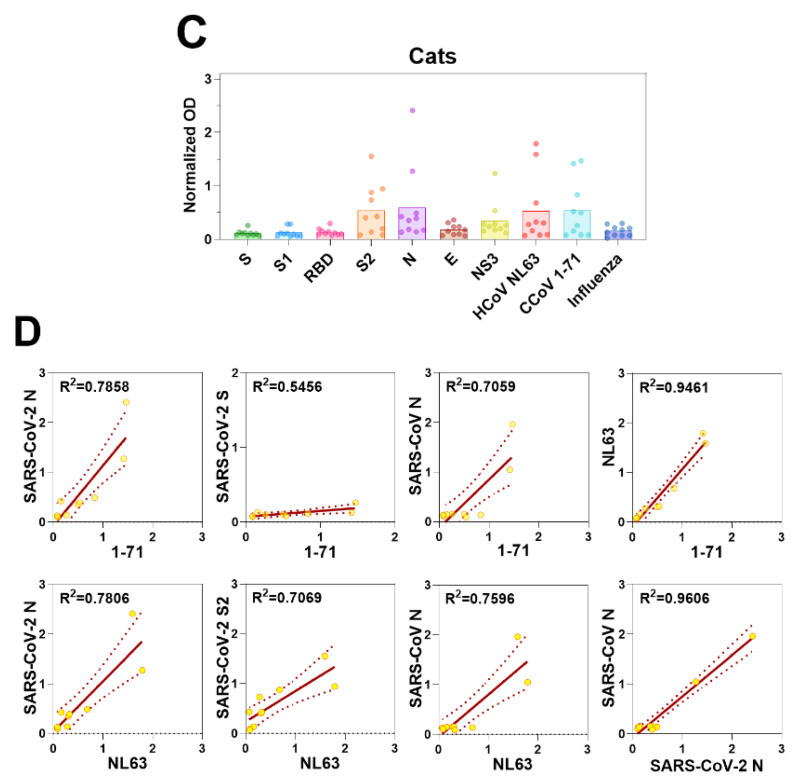
Antibody responses in dogs and cats. (**A**) Serum antibody responses of dogs to S, S1, RBD, S2, N, E, and NS3 viral proteins of SARS-CoV-2, and inactivated viruses, CCoV 1-71, HCoV NL63, and H1N1 influenza virus were measured by IgG ELISA. The OD values were normalized by subtracting the OD values generated by sera from SPF dogs. (**B**) In canine serum samples, IgG levels against CCoV/HCoV and those against S, S1, RBD, S2, N, E, and NS3 of SARS-CoV-2, as detected by ELISA, were used in correlation analyses. (**C**) Serum IgG antibody responses of cats to SARS-CoV-2 S, S1, RBD, S2, N, E, and NS3, and and inactivated viruses, CCoV 1-71, HCoV NL63, and H1N1 influenza virus were measured by ELISA. The crude OD values generated by ELISA were normalized by subtracting the OD values generated by sera from SPF cats. (**D**) ELISA was also used to determine IgG antibody levels against N of SARS-CoV-2, CCoV 1-71, and HCoV NL63 in the sera of cats for correlation analyses.

We did not detect antibodies specific to SARS-CoV-2 or other coronaviruses in the sera of the SPF dogs (data not shown). Some dogs from the veterinary clinic, however, had detectable levels of antibodies to CCoV 1-71, HCoV NL63, and influenza A virus, as well as antibodies to viral proteins of SARS-CoV-2, S2 and N in particular (Figure 4A). The animals that had the highest responses to SARS-CoV-2 S2 and N proteins were also those that had the highest levels of reactivity to the inactivated CCoV 1-71 and HCoV NL63 viruses. This result is consistent with the cross reactivity of SARS-CoV-2 with CCoV 1-71 and HCoV NL63 in the murine sera and provides a warning against overinterpreting SARS-CoV-2 seroepidemiology studies in dogs.

We further investigated the relations between antibody responses to CCoV 1-71 or HCoV NL63 and those to SARS-CoV-2 by using the 23 canine serum samples that showed the highest antibody titers to CCoV 1-71 and HCoV NL63. Antibody titers to CCoV 1-71 were positively correlated with those to SARS-CoV-2 N (95% CI, 0.4694–0.289; R^2^ = 0.4866) and S2 (95% CI, 0.06348–1.18; R^2^ = 0.2035), and antibody titers to HCoV NL63 showed a good correlation with SARS-CoV-2 N (95% CI, 0.7029–1.564; R^2^ = 0.5753) and NS3 (95% CI, 0.3071–0.4514; R^2^ = 0.8437) (Figure 4B).

We also assessed the reactivity of serum samples from 13 domestic cats, including 2 with confirmed FIP and 3 SPF cats included as negative controls. No coronavirus antibodies were detected in the sera from the SPF animals (data not shown). Sera from cats previously infected with FIP reacted strongly to HCoV NL63, CCoV 1-71, SARS-CoV N, and SARS-CoV-2 N and S2 proteins. However, they reacted weakly to SARS-CoV S and SARS-CoV-2 S1 and RBD proteins (Figure 4C and D). As seen with canine samples, some serum samples taken from cats at the veterinary clinic had reactivity to SARS-CoV-2 proteins (N, S2, and NS3) (Figure 4C). These same sera also had strongest reactivity to HCoV NL63 and CCoV 1-71. Taken together with the lack of S, S1, or RBD reactivity suggests the SARS-CoV-2 reactivity was due to cross reactivity and not prior exposure.

## 4. Discussion

A detailed understanding of the antibody response to SARS-CoV-2 is essential for many facets of developing a vaccine in response to the COVID-19 pandemic. Here, we examined several measures of SARS-CoV-2 antibody responses with an emphasis on cross-reactivity and individual protein-specific responses. Thirty-five individuals had detectable IgG levels against SARS-CoV-2 S, S1, RBD, S2, N, E and NS3-specific antibodies, with RBD titers correlating best with neutralizing activity. In contrast, mice immunized with γ-irradiated SARS-CoV-2 virus developed poor responses against most of the same proteins, with a dominance of response noted to S2. Why there is a difference between natural infection and immunization responses is unclear, but it could be caused by variables such as host, antigen dose, and/or differential presentation of antigens driven by active virus replication following natural infection. The mechanisms underlying these differential responses are potentially of more than just academic interest. Although antibody-driven immunity is believed to restrict virus infection, there have been reports of coronavirus reinfection with 229E, HKU1, and OC43 [18,19] and an emerging report of the same phenomenon with SARS-CoV-2 [11]. Conversely and paradoxically, the presence of specific antibodies can accelerate virus infection and exacerbate the disease, including in SARS-CoV models [12,20]. Immunization of cats against FIP can increase the disease severity through antibody-dependent enhancement [21]. In such systems, enhancement of disease is often driven by non-neutralizing antibody responses [22]. Therefore, understanding the factors that drive the RBD-specific neutralizing responses may better inform the development of safer, more effective COVID-19 vaccines. It should also be noted that we detected neutralizing responses in only 45% of individuals.

The presence of related endemic HCoVs raises the possibility that prior infection with these viruses may influence the nature and/or kinetics of the antibody response to SARS-CoV-2. This is best illustrated in the influenza field, where there is a growing understanding that an individual’s first influenza virus infection can modulate the response to viruses encountered decades later [23]. We detected various levels of antibody to 229E, NL63, OC43, and HKU1 in our small cohort; however, these levels did not correlate well with the SARS-CoV-2 antibody responses. This finding suggests that prior HCoV exposure does not prime for a more robust SARS-CoV-2 response and, by inference, does not modulate the severity of COVID-19 disease. The lack of SARS-CoV-2 reactivity of negative control sera is also consistent with this.

We determined the baseline levels of antibodies to other common respiratory viruses to which all of the individuals should have been exposed. High antibody levels were detected to RSV A (LA2-94/2013) and B (TH-10526/2014) fusion proteins and influenza A and B virus hemagglutinin proteins. Variable responses were seen to other viruses, including HCoV and MNV. Although exact exposure histories were unknown, these data suggest a waning of antibody titers to at least some respiratory viruses is to be expected. This phenomenon has received much attention in the context of COVID-19, and some have suggested a short duration of responding antibodies [6,24,25]. This has been interpreted to mean a short duration of vaccine-mediated protection and likelihood of reinfection. Of note, the protein microarray that we used contained AdV antigens (types 3,4,7). AdVs have been used as backbone vectors for multiple COVID-19 vaccine approaches [26,27,28], and preexisting antibodies to the AdV5 version of the vector have led to suboptimal responses to COVID-19 vaccines [28]. To avoid the AdV5 issue, vaccine developers have chosen alternatives: Astrazeneca PLC uses a chimpanzee AdV [26], and Johnson & Johnson uses a rarer strain, AdV26 [27]. It remains to be seen whether any preexisting seasonal AdV immunity will modulate the effectiveness of these vaccines.

Soon after the pandemic virus was identified, reports emerged of natural or experimental infections in cats and dogs [29,30]. These reports were supported by computational predictions of potential host range [31]. Due to these reports and some of the cross-reactivities we observed in immunized murine sera, we decided to explore potential cross-reactivities in sera from these companion animals. Antibody responses of cats were highly correlated between SARS-CoV-2 and HCoV NL63, and SARS-CoV-2 and CCoV 1-71, particularly in responses with N proteins. Although FIP is a relatively rare outcome of FCoV, approximately 10% of cats are infected with FCoV [32]. Taken with the level of reactivity we found to SARS-CoV-2 N proteins in the canine sera, the feline sera analysis indicated that seroprevalence studies in these hosts should be interpreted with caution. Baseline reactivity to SARS-CoV-2 will create a degree of false positivity if SARS-CoV-2 antigens are used alone; thus, other antigens or unexposed control samples need to be included. Similarly, properly validating ELISAs for SARS-CoV-2 antigens using a large number of negative sera samples from cats or dogs is required to determine the specificity of any ELISAs employed.

There is still much to learn about the virus/host interaction involving SARS-CoV-2, but the speed at which scientific data are being accumulated is impressive. Multiple vaccines have entered advanced stage clinical trials within 8 months of the virus sequence being shared, and studies to examine the incidence of infection are underway. Our data suggest that the antibody response to SARS-CoV-2 varies across individuals and with a magnitude seemingly independent of antibody levels to other coronaviruses. While our data are convincing in this regard, we do acknowledge the relatively small sample size. Expanding our work to larger panels of sera with more associated metadata may find some patterns within this observed variation and should, indeed, be a focus of future work. 

## 5. Conclusions

Vaccines are designed to induce protective immune responses to a specific infection. A successful COVID-19 vaccine should elicit protective antibodies while eliminating potential disease-enhancing antibody effects. We characterized antibodies in the serum of patients who had recovered from COVID-19 and could show variability in individual SARS-CoV-2-specific responses. Serum from individuals with high levels of RBD-specific antibodies were more able to neutralize the virus. Other non-SARS-CoV respiratory virus-specific antibody responses in humans were not relevant to the SARS-CoV-2 viral protein-specific antibodies. Preexisting cross-reactive antibodies to respiratory viruses in animals were strongly associated with SARS-CoV-2 N or S2-specific antibodies; however, those antibodies were not able to neutralize the virus. Our findings stress the need to test multiple vaccine platforms as rapidly as possible and to account for the expected interperson variability.
